# Machine Learning-Driven Risk Prediction Models for Posthepatectomy Liver Failure: A Narrative Review

**DOI:** 10.3390/medicina62020237

**Published:** 2026-01-23

**Authors:** Ioannis Margaris, Maria Papadoliopoulou, Periklis G. Foukas, Konstantinos Festas, Aphrodite Fotiadou, Apostolos E. Papalois, Nikolaos Arkadopoulos, Ioannis Hatzaras

**Affiliations:** 1 Eugenideio Hospital, National and Kapodistrian University of Athens, 11528 Athens, Greece; 2 4th Department of Surgery, Attikon University Hospital, School of Medicine, National and Kapodistrian University of Athens, 12462 Athens, Greece; mpapadoliop@med.uoa.gr (M.P.); narkadopoulos@med.uoa.gr (N.A.); ihatzaras@med.uoa.gr (I.H.); 3 2nd Department of Pathology, Attikon University Hospital, School of Medicine, National and Kapodistrian University of Athens, 12462 Athens, Greece; pfoukas@med.uoa.gr (P.G.F.); kosfes@hotmail.gr (K.F.); 4 Department of General, Visceral, and Transplantation Surgery, Heidelberg University Hospital, Ruprecht Karl University of Heidelberg, 69120 Heidelberg, Germany; afroditi.fotiadou@med.uni-heidelberg.de; 5 Hellenic Society of Gastrointestinal Oncology, 12461 Athens, Greece; apapalois@med.uoa.gr; 6 2nd Department of Surgery, Aretaieion University Hospital, School of Medicine, National and Kapodistrian University of Athens, 11528 Athens, Greece

**Keywords:** machine learning, artificial intelligence, prediction models, posthepatectomy liver failure, liver resection

## Abstract

*Background and Objectives*: Posthepatectomy liver failure (PHLF) remains a major cause of morbidity and mortality for patients undergoing major liver resections. Recent research highlights the expanding role of machine learning (ML), a crucial subfield of artificial intelligence (AI), in optimizing risk stratification. The aim of the current study was to review, elaborate on and critically analyze the available literature regarding the use of ML-driven risk prediction models for posthepatectomy liver failure. *Materials and Methods*: A systematic search was conducted in the PubMed/MEDLINE, Scopus and Web of Science databases. Fifteen studies that trained and validated ML models for prediction of PHLF were further included and analyzed. *Results*: The available literature supports the value of ML-derived models for PHLF prediction. Perioperative clinical, laboratory and imaging features have been combined in a variety of different algorithms to provided interpretable and accurate models for identifying patients at risk of PHLF. The ML-based algorithms have consistently demonstrated high area under the curve and sensitivity values, surpassing traditionally used risk scores in predictive performance. Limitations include the small sample sizes, heterogeneity in populations included, lack of external validation and a reported poor ability to distinguish between true positive and false positive cases in several studies. *Conclusions*: Despite the constraints, ML-driven tools, in combination with traditional scoring systems and clinical insight, may enable early and accurate PHLF risk detection, personalized surgical planning and optimization of postoperative outcomes in liver surgery.

## 1. Introduction

Liver resection remains the cornerstone of treatment for most of the primary and secondary malignant neoplasms of the liver. With recent technological advances and a better understanding of the physiological processes implicated in liver regeneration, hepatectomy can be performed in appropriately selected patients with minimal morbidity and mortality. However, especially in cases of extended resections, there is an underlying risk for posthepatectomy liver failure (PHLF). A resultant small liver remnant and a diseased parenchyma, secondary to cirrhosis or hepatotoxic chemotherapy, are well-established risk factors leading to an acquired postoperative deterioration in the liver’s ability to adequately perform its synthetic, excretory and detoxifying functions [1].

PHLF incidence varies in the literature and can be as high as 32% with a related mortality of up to 54% [2,3]. In the past, there was no uniformity in defining PHLF, and the 50-50 criterion was proposed; alternatively, a peak postoperative bilirubin of 7.0 mg/dL was considered a sensitive and specific marker of posthepatectomy liver failure-related deaths [2,4]. The International Study Group of Liver Surgery (ISGLS) provided us with a definition of PHLF, encompassing abnormal INR and hyperbilirubinemia observed on or after the fifth postoperative day, as well as a grading system, which has been widely accepted and utilized in daily practice by liver surgeons [3].

In general, the risk of PHLF is mainly driven by the quantity as well as the quality of liver parenchyma after the operation. Several traditional scoring systems, such as the Child–Pugh score, model for end-stage liver disease (MELD) score, albumin–bilirubin (ALBI) score and fibrosis-4 (FIB-4) index, have been extensively used for the early prediction of PHLF, albeit with several limitations which may limit their generalizability and overall predictive performance [5,6,7]. Similarly, three-dimensional image reconstruction techniques have enabled accurate measurements of postoperative liver remnant volumes. While the aforementioned values have been associated with the risk of developing serious postoperative complications, relying solely on volumetric estimations neglects the assessment of the underlying liver quality, thereby limiting their application in patients with damaged livers [8,9].

Machine learning (ML) is a relatively new and promising subfield of artificial intelligence (AI). It has the ability to harness large datasets of electronic health records and imaging features and develop accurate prediction models. The speed and complexity of analysis highlight its potential to identify previously unrecognized relations and enhance risk stratification, surgical planning and outcomes [10]. To this end, several different ML models have been used, including tree-based algorithms, random forests and complex artificial neural networks. Their performance is commonly assessed by using metrics including the area under the receiver operating characteristic curve (AUC), accuracy, sensitivity, specificity and predictive values. To enhance the interpretability of the resultant risk prediction tools, techniques including the SHapley Additive exPlanations (SHAP) analysis are able to graphically identify the most influential predictors. The aim of the current study was to review, elaborate on and critically analyze the available literature regarding the use of ML-driven risk prediction models for posthepatectomy liver failure.

## 2. Materials and Methods

### 2.1. Search Strategy

A systematic search was conducted in the PubMed/MEDLINE, Scopus and Web of Science Core Collection databases by using Medical Subject Headings (MeSH) and keywords combined with Boolean operators. The last electronic search was conducted in January 2026. The complete search strategy is available in Supplementary Table S1. Reference lists from the included studies were also manually screened to identify eligible studies.

### 2.2. Inclusion Criteria

We included original studies that trained and validated machine learning (ML) models for prediction of posthepatectomy liver failure (PHLF). The population included patients who underwent hepatectomy, irrespective of etiology. Eligible studies applied ML algorithms—including but not limited to random forests, gradient boosting machines, artificial neural networks and deep learning models—to predict PHLF using perioperative clinical, biochemical and imaging data. Studies were required to report model performance using metrics such as the AUC, sensitivity, specificity and predictive values.

### 2.3. Exclusion Criteria

We excluded studies that used AI/ML only for feature extraction (for example, image processing and radiomics extraction from liver segmentation), feature selection, feature grouping or PHLF clustering, without devising a predictive model for PHLF. Additionally, studies that did not report internal validation of the prediction model (for example, bootstrapping, cross-validation or split-sample techniques) or with inadequate methodological details were excluded. Finally, classical statistical models, such as regression analysis models, were also excluded.

Search strategy, article selection and data extraction were assessed by two independent authors. Any areas of disagreement were resolved through a case-by-case discussion. Due to the heterogeneity of the included studies’ populations, methodology and reported results, a narrative synthesis approach was selected.

## 3. Results

**Figure 1:**
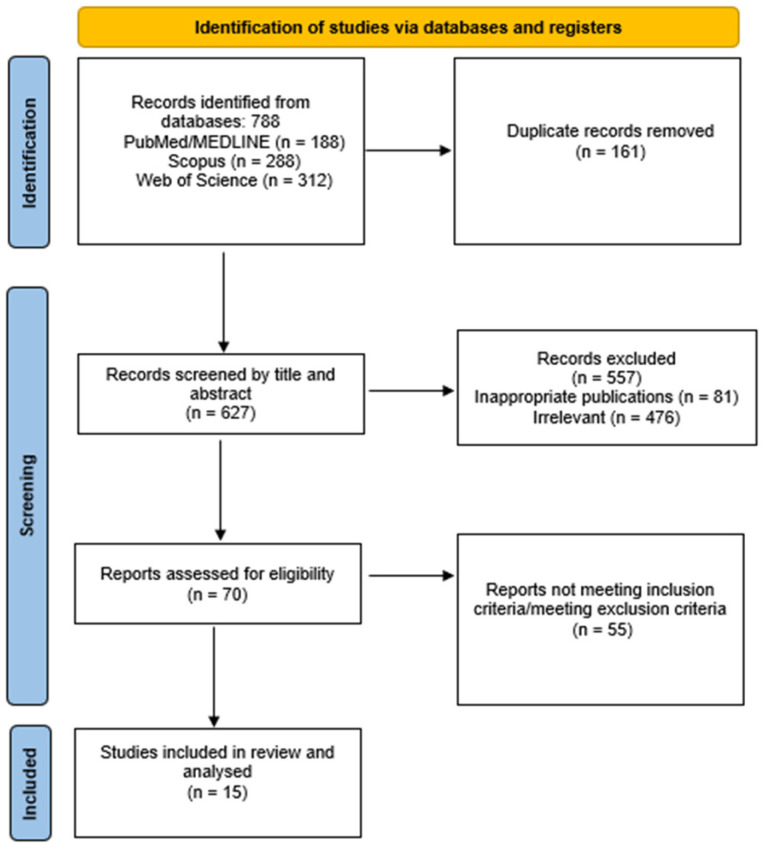
Flowchart of the study selection process.

The literature search identified 627 unique studies, which were further assessed by title and abstract screening for eligibility. Five hundred fifty-seven articles were excluded as irrelevant or as inappropriate publication types (case reports, editorials, review articles, experimental studies, errata or Congress proceedings). Following full-text screening, 15 studies were finally included and analyzed in this review [11,12,13,14,15,16,17,18,19,20,21,22,23,24,25]. The study selection process is depicted in Figure 1.

Table 1 summarizes characteristics of the included studies. Most of them were retrospective in design, single or multi-institutional studies, with a variable number of participants ranging from 226 to 28,192 patients.

The majority of them used preoperative clinical and biochemical features, as well as radiological volumetric indices and intraoperative parameters, to train, tune and test ML algorithms for the prediction of PHLF.

### 3.1. Artificial Neural Network-Based Models

The seminal article by Mai et al. [25] included 353 treatment-naïve patients with hepatocellular carcinoma (HCC), who underwent hemihepatectomy. The objective was to construct and validate an artificial neural network (ANN) for prediction of severe PHLF (grades B–C), which occurred in 25% of the study population. Five independent risk factors were identified by multivariable logistic regression analysis and were included to establish the ANN model, which demonstrated an AUC of 0.880 (95% confidence interval [CI]: 0.836–0.925) and 0.876 (95% CI: 0.801–0.950) in the development and validation sets, respectively. It displayed a significantly better discriminative ability compared to the traditional logistic regression model, with the commonly used clinical scores including the Child–Pugh, MELD and ALBI scores and indocyanine green retention test at 15 min (ICGR15). Superiority was confirmed in all subgroup analyses performed. The three most important risk factors identified were the standardized future liver remnant (sFLR), total bilirubin (TBil) and platelet count (PLT). It is of note that 11% of the patients with severe PHLF had an sFLR of approximately 70%, reiterating the notion that relying solely on volumetric estimation of the post-resection liver quantity is insufficient to predict the occurrence of PHLF. Despite the high discriminative ability of the model, the positive predictive value (PPV) was only 45.51% in the validation set, with a sensitivity of 95.2%, a specificity of 65.7% and a negative predictive value (NPV) of 97.78%.

A recent study by Jin et al. [17] also devised preoperative and postoperative ANN algorithms to predict severe PHLF, demonstrating good predictive performance, which outperformed the traditional clinical risk scores. The authors also used an external validation cohort consisting of 31 patients, in which the preoperative and postoperative models’ AUCs were 0.720 and 0.732, respectively. Nonetheless, reported precision was low, signifying low confidence in the positive predictions. Another study integrated clinically significant portal hypertension in the construction of an ANN model, in an effort to select appropriate surgical candidates among patients with HCC and portal hypertension [23]. The authors reported a better discriminatory performance and a superior net benefit of the model compared to conventional scores.

In a large recently published multi-institutional study including 1832 patients, a deep learning Bidirectional Encoder Representations from Transformers (BERT)-based model accurately predicted PHLF early within the first 24 h after the operation by exploiting perioperative electronic health records and temporal differences between the preoperative and postoperative stages [15]. The model achieved an AUC of 0.952 (95% CI 0.907–0.984) in the internal validation cohort, 0.884 (95% CI 0.849–0.915) in the combined (Chinese) external validation cohort and 0.654 (95% CI 0.558–0.760) in the external Western cohort (with largely incomplete perioperative data). In all cases, the model proved to be superior in terms of predictive performance when compared to other commonly used machine learning models and algorithms. According to SHAP analysis, the top three most important features were postoperative day 1 (POD1) prothrombin time (PT)/international normalized ratio (INR), number of resected segments and hepatitis B (HBV) infection. For predicting clinically relevant PHLF in the combined Chinese validation group of patients, the sensitivity of the model was approximately 85%, correctly identifying 23 out of 27 patients with PHLF grades B–C. The versatility of the model proved to be significant through scrutinized subsequent analyses including subpopulation analyses, analyses with restriction of inputs to preoperative and anticipated intraoperative data, analyses with incomplete variable inputs, and risk stratification grouping of patients. Furthermore, the predictions of the clinicians markedly improved with the use of the constructed model.

### 3.2. Tree-Based Ensemble Boosting Models

Three studies used light gradient boosting machine (LightGBM), which is an open-source machine learning framework developed by Microsoft Corporation (Redmond, WA, USA) characterized by fast training speeds and low memory usage [12,16,24]. They collectively showed AUCs ranging from 0.703 to 0.870 following cross-validation or by using an independent testing cohort of patients. In a multicenter retrospective study including 875 HCC patients, the LightGBM-based model demonstrated higher diagnostic performance when compared to traditional scoring systems (AUCs in the test set of 0.822 versus 0.703, 0.619, 0.613, 0.574 and 0.549 for the ML model versus the ALBI, FIB-4, AST to Platelet Ratio Index (APRI), MELD and Child–Pugh scores, respectively) and added clinical value [24]. Sensitivity, specificity, PPV, NPV and accuracy were 87.5%, 64.4%, 42.2%, 94.6% and 69.7%, respectively. Recently, a risk prediction model was developed and validated incorporating the controlled nutritional status score (CONUT) in HCC patients, which has been previously established as a predictor of survival and complications in several solid organ malignancies [16,26]. In the aforementioned study, the CONUT score had the greatest impact on the occurrence of PHLF [16]. Furthermore, patients with a high (≥4) CONUT score were more likely to suffer from portal hypertension and liver cirrhosis, more likely to need intraoperative transfusions and experience a prolonged hospital stay, and more likely to develop clinically significant PHLF (22% versus 4%, *p* = 0.019). The authors suggested that such a high CONUT score might be an indication for early nutritional support and optimization of surgical patients. Finally, Nair et al. harvested electronic health records data from the large National Surgical Quality Improvement Program (NSQIP), which included 28,192 patients who underwent elective hepatectomy irrespective of indication [12]. Among the different ML models evaluated, LightGBM performed the best across all cross-validation runs. According to SHAP analysis, the five most influential risk factors were transfusions, right lobectomy, preoperative INR, preoperative sodium (Na) level and operative time. Setting a threshold of PHLF probability at 5%, INR levels above 1.04 and Na levels below 139 mEq/L were associated with a high risk. Therefore, the authors emphasized that results should be viewed cautiously and primarily as an indication of how ML-based algorithms may detect subtle biochemical changes increasing the risk for PHLF. Additional limitations are relevant to large population studies and the fact that the NSQIP database lacks clinical insight, for example, in differentiating the grades of PHLF.

Extreme gradient boosting (XGBoost) is yet another machine learning model that has been tested in its ability to predict PHLF occurrence. While being a tree-based gradient boosting framework, similar to LightGBM, it is conceptually different, slower in large datasets, yet stable and reliable. In an effort to construct a reliable PHLF prediction model for patients who underwent first-time hepatectomy for a primary liver cancer (HCC), the 12 best variables were identified and 12 ML models were evaluated [14]. The XGBoost model was found to avoid overfitting, with its predictive accuracy decreasing with the increase in the proportion of the training set and increasing in the validation set. This model exhibited an AUC of 0.983, 0.981 and 0.942 in the training, validation and prospective validation cohorts, respectively, outperforming the traditional risk scores. Decision curve analysis (DCA) confirmed a high net clinical benefit within a range of probabilities. Another single-center study included 334 patients with primary and metastatic liver cancer and used Pycaret, an open-source library in Python that included 15 different ML classification models [18]. The XGBoost model was the best model. Nonetheless, despite the fact that the algorithm effectively predicted negative PHLF cases, its predictive performance for positive PHLF cases was inadequate. Specifically, the model predicted only five out of nine cases of PHLF in the test set, yielding a sensitivity of 0.556, specificity of 0.989, precision (PPV) of 0.833 and NPV of 0.958. Its overall poor performance in predicting PHLF can be attributed to class imbalance due to a low PHLF incidence of approximately 9% in this study.

Recently, tree-based architectures predicting PHLF occurrence incorporating molecular liver regeneration biomarkers have been developed [13]. The researchers introduced and exploited indices of liver regeneration into their predictive models, based on the RAMP2/GATA3-VEGFA/PEDF signaling pathway. Most importantly, they reported on a prediction framework encompassing preoperative and intraoperative models, which achieves an exceptional class-specific precision in patients identified as consensus high-risk (94.4–96.6% precision for PHLF) and consensus low-risk (92.1–95.5% for non-PHLF). Despite the fact that the latter classification and reported metrics were applicable to approximately 70–80% of included patients, the results are extremely helpful for clinicians aiming to detect PHLF at an early stage and initiate appropriate interventions.

### 3.3. The Use of Radiomics in Model Construction and Evaluation

Radiomics refers to the emerging field of processing and extracting imaging features, including data related to shape, texture and signal intensity, derived from commonly used radiological modalities, such as computed tomography (CT) and magnetic resonance imaging (MRI) scans or ultrasound [27]. Radiomics data, invisible to the human eye, can then be used to devise accurate prediction models.

Five of the herein-included studies extracted and utilized radiomics features to build a PHLF machine learning-based prediction algorithm [11,19,20,21,22]. In a multi-institutional Italian study involving 500 HCC patients operated on with curative intent, nineteen radiomics and five clinical features were selected based on principal component analysis and univariable and multivariable logistic regression analysis to build three predictive models [11]. Considering calibration and discrimination, an average external ensemble model (AEM), including the XGBoost and Random Forest (RF) models, was built with an AUC of 90.10% in the test set and 83.9% (IQR 76.7–92.3) after bootstrapping. With a cut-off point at 0.078, the sensitivity was 80%, specificity 89.5%, PPV 21%, and NPV 99.2%. Of the clinical factors, only cirrhosis was included among the five most important risk features.

In another European study, radiomics features from a “virtual biopsy” portal phase CT of a non-tumorous liver region significantly improved the prediction of liver dysfunction [20]. Even though traditional clinical predictors were still retained, the addition of textural features boosted the model’s discrimination ability.

Zhong et al. built a clinical–radiomics combination prediction model for symptomatic (clinically significant) PHLF, based on imaging features from two-dimensional shear wave elastography (2D-SWE) [21]. The Random Forest classifier-based clinical–radiomics model achieved an AUC of 0.822 (95% CI 0.720–0.898) in the test cohort. Accuracy, sensitivity, specificity, PPV and NPV were 0.750, 0.704, 0.773, 0.612 and 0.836, respectively, in the test set. The model’s diagnostic performance was significantly better than the MELD and ALBI scores’ performances and outperformed the clinical-only and radiomics-only models. According to SHAP analysis, first-order radiomics features, corresponding to higher liver stiffness, were among the three most influential variables of the model.

Convolutional neural networks (CNNs) are a form of specialized deep learning models that are particularly useful for the processing and utilization of grid-like data like images. Xue et al. built a PHLF prediction model by combining clinical features and radiomics features stemming from dual-modal ultrasound [19]. The authors designed a custom-made prediction model (PHLF-net) using ResNet50 (a 50-layer deep convolutional neural network) as a backbone, by incorporating conventional B-mode images and elastography images and concatenating clinical indices. The reported metrics of the PHLF-net model in the internal independent testing cohort were AUC 0.923 (95% CI 0.838–0.971), sensitivity 82.1%, specificity 95.8%, PPV 92%, NPV 90.2%, positive likelihood ratio (LR+) 19.7 and negative likelihood ratio (LR−) 0.2. The respective metrics in the three external testing cohorts reproduced the significant diagnostic performance of the model. Similarly, in a study involving 265 patients who underwent hemihepatectomy irrespective of etiology (including benign and malignant cases), the authors used a 3D DenseNet-based convolutional neural network and were able to merge low- and high-level CT image features to predict the occurrence of PHLF [22]. In 5-fold cross-validation, the deep learning model performed with an AUC of 0.7927, accuracy of 84.15%, sensitivity of 71.43% and specificity of 89.66%.

## 4. Discussion

Breakthroughs in artificial intelligence have revolutionized patient care across several medical settings, including hepatic surgery. Liver resection is the mainstay of treatment for appropriate candidates presenting with localized primary liver tumors or even for malignancies metastatic to the liver. However, PHLF remains the major cause of postoperative mortality and has also been associated with a prolongation of hospital stays, increased costs and poor overall survival [4,28,29]. In this setting, early detection of high-risk patients may have a significant impact on preoperative planning, intraoperative decision-making and postoperative vigilance, promoting evidence-based personalized medicine and enhancing patient outcomes [30].

Even though traditional clinical scores have been extensively used in the past, their performance suffers from significant limitations, including subjectivity in assessment of clinical indices, poor performance and lack of generalizability [5,6,7]. The theoretical advantages of ML-driven models are the ability to process and analyze large datasets and to detect subtle and non-linear relationships, allowing formulation of algorithms considering all possible interactions between variables [24]. Collectively, our study results underscore the importance of machine learning models in the early and accurate detection of PHLF. This was highlighted in the overall good predictive performance of all models used across the studies, with AUC values commonly exceeding 80%, good reported calibration and added clinical benefits on decision curve analyses. Whenever a comparison was made to traditional indexes, including the MELD, ALBI and Child–Pugh scores, the ML models had a significantly better predictive performance. Decision curve analysis provides a graphical representation of net clinical benefit over various thresholds of risk probability, substantially complementing a model’s predictive performance metrics. It balances between the identification of true positive cases (patients who were correctly identified and ended up developing PHLF) and the avoidance of false positive cases (in this scenario, patients who were falsely identified as high-risk, when they did not actually end up developing PHLF), capturing clinical usefulness. From the available data in this review, ML-driven models’ net clinical benefit has remained high across a range of thresholds, surpassing several traditional risk prediction models [13,14,16,21,23,24]. On the other hand, statistical limitations, including class imbalance and model optimization to maximize sensitivity, were reflected in the poor ability to distinguish between true positive and false positive cases. Indeed, in several studies, the resultant models suffered from low precision and/or specificity [11,17,24,25], signifying that many predicted high-risk patients will not eventually develop PHLF. The aforementioned results imply that ML models should preferentially be used for screening high-risk patients, rather than as a uniformly applied decision-making tool. The latter also stresses the importance of physicians’ judgment, stemming from clinical experience—load of cases, knowledge and reasoning. Hence, these novel and groundbreaking tools should be used with caution and as evaluations complementary to the physician’s critical thinking, as well as to traditional and validated risk assessment tools with lasting value over time.

The integration of ML-derived PHLF risk prediction models into everyday clinical practice remains challenging and, so far, exploratory. Ideally, in a preoperative clinical setting, electronic health records, including baseline clinical and biochemical parameters, would be automatically inserted into a prospective institutional database and provided to the model. Manual input may be required for semi-automatic radiomics feature extraction or planned surgical procedures and resultant liver reserve. Preoperative detection of high-risk patients would enhance informed decision-making. Additionally, surgeons could opt for a risk reduction strategy, including portal vein embolization or a parenchyma-sparing procedure, while also correctly allocating intensive care unit beds and promoting a stringent postoperative surveillance strategy. During the postoperative course, the model would be continuously updated with intraoperative and postoperative values, boosting its predictive accuracy and enabling even more precise and patient-tailored risk estimation, eventually leading to preemptive measures mitigating the risk of developing clinically relevant PHLF.

A variety of different ML models were used. LightGBM, XGBoost, Random Forest, ANNs and CNNs were all employed. These models differ substantially in their underlying architectures, learning processes and interpretability. Tree-based methods seem to handle clinical and biochemical data well and provide interpretable, clinically useful models. On the other hand, artificial neural networks and specialized convolutional neural networks are extremely useful in harnessing large datasets and imaging features, but suffer from poor interpretability, also referred to as the “black box” phenomenon [31,32]. However, several studies have tried to combat this caveat and enhance the interpretability of their model by using SHAP analysis. Stemming from game theory, Shapley explanations provide a contribution analysis of each feature to the final prediction model. Hence, readers and fellow researchers can assess how much and in which direction individual variables may affect the final prediction. One can also argue that a “hybrid” technique, by which ML can be used for feature mining (with SHAP value identification of the most influential variables), which will then be used to guide simple prediction rules or thresholds, can be exploited to proactively develop easy-to-use and interpretable tools. Most of the studies used preoperative and intraoperative clinical and biochemical variables, with few studies encompassing radiomics features to train and validate their prediction model. From the available evidence on SHAP analysis, there was also marked variability in the reported most influential factors affecting the prediction. Several well-known traditional risk factors have been consistently identified, including preoperative bilirubin levels, INR, PLTs and the extent of resection. However, depending on individual studies’ methodology and availability of data, other factors, such as creatinine, first-order radiomics and clinical and nutritional indices, have been invariably reported.

### Sources of Heterogeneity and Constraints on Generalizability

Most of the included studies were retrospective in design and had a small number of participants. Importantly, only five studies used an independent testing cohort for external validation of their model [13,15,17,19,23]. In most cases, a decline in model performance was observed during external validation. Furthermore, two out of the five previously mentioned studies relied on very small external validation cohorts, with sample sizes not exceeding 31 patients. Additionally, only four of the analyzed studies included hepatectomized patients from a Western population [11,12,15,20]. Of the remaining studies, ten originated from China and one from Japan; hence, the inclusion of predominantly Eastern populations may hamper generalizability to other populations, not due to machine learning inherent deficiencies, but rather due to different underlying physiological and pathophysiological processes. The latter also explains the high rates of HBV-related HCC patients included, while other etiologic factors for cirrhosis and development of HCC, including alcoholic steatohepatitis and non-alcoholic steatohepatitis, may have been underrepresented. Finally, there was significant heterogeneity among studies with regard to PHLF outcomes examined (all grades versus clinically significant PHLF), the extent of liver resection performed (minor versus major), underlying pathology (benign versus malignant cases) and baseline status of the patients included (treatment-naïve patients versus patients who had received preoperative systematic or ablative treatments versus patients who had undergone a repeat hepatectomy).

## 5. Conclusions

Despite the obvious constraints, we believe that there is a growing amount of recently published evidence supporting the role of machine learning algorithms in the prediction of PHLF. Machine learning-driven tools, in combination with traditional scoring systems and clinical insight, may enable early and accurate risk detection, personalized surgical planning and optimization of postoperative outcomes. Further large-scale and well-designed prospective studies, which include patients from Western populations and incorporate external validation cohorts, are needed to strengthen the current level of evidence.

## Figures and Tables

**Table 1:** Characteristics of the included studies.

Author (Year)	Study Design	Number of Patients	Indications	ML Model	Performance in Internal Validation and Testing Cohorts	Most Influential Factors	Performance in External Validation Cohorts
Famularo (2025) [11]	Retrospective multi-institutional	500	Curative-intent hepatectomy for HCC	Average ensemble model (XGBoost + RF)	AUC 0.901 and 83.9% (IQR 76.7–92.3) after bootstrapping, sensitivity 80%, specificity 89.5%, PPV 21%, NPV 99.2%	Cirrhosis and radiomics features	-
Nair (2025) [12]	Retrospective (NSQIP database)	28,192	Hepatectomy for any indication	LightGBM	AUC 0.8349 (95% CI 0.8272–0.8427)	Transfusions, right lobectomy, preop INR	-
Shen (2025) [13]	Retrospective multi-institutional	1071	Major hepatectomy	LightGBM, XGBoost	Preoperative model AUC 0.754 (95% CI 0.717–0.790); Intraoperative model AUC 0.787 (95% CI 0.728–0.846);	Different per model pre/intra/postoperative	AUCs 0.740–0.895
					Postoperative model AUC 0.904 (95%CI 0.883–0.924)		
Tang (2025) [14]	Retrospective single centre (+prospective test cohort)	312	First-time hepatectomy for HCC	XGBoost	AUC 0.981; AUC 0.942 (prospective cohort)	TBil, MELD, ICGR15	-
Wang (2025) [15]	Retrospective multi-institutional	1832	Treatment-naïve hepatectomized patients	BERT-based	AUC 0.952 (95% CI 0.907–0.984), sensitivity 0.692, specificity 0.962, PPV 0.6, NPV 0.974	POD1 PT/INR, number resected segments, HBV	AUC 0.884 (95% CI 0.849–0.915), sensitivity 0.747, specificity 0.842, PPV 0.294, NPV 0.974 in the combined external Chinese validation cohort;
							AUC 0.654 (95% CI 0.558–0.760), sensitivity 0.6, specificity 0.668, PPV 0.172, NPV 0.935 in the Western cohort
Yuan (2025) [16]	Retrospective single centre	464	HCC patients	LightGBM	Test group: AUC 0.703 (95% CI 0.528–0.879); Validation group: AUC 0.808 (95% CI 0.637–0.980)	CONUT, INR, AST to neutrophil ratio index (ANRI)	-
Jin (2024) [17]	Retrospective single centre	226	Therapeutic hepatectomy	ANN	Preoperative model AUC 0.766 (95% CI 0.752–0.781); Postoperative model AUC 0.851(95% CI 0.838–0.864)	Child–Pugh, Tbil, Creatinine	AUCs of 0.720 and 0.732 for preoperative and postoperative models, respectively
Tashiro (2024) [18]	Retrospective single centre	334	Primary and metastatic liver cancer	XGBoost	Sensitivity 0.556, specificity 0.989, PPV 0.833, NPV 0.958	Liver resection rate (Res), albumin, PT	-
Xue (2024) [19]	Prospective multi-institutional	532	HBV-related HCC patients	ResNet50-based CNN	Internal validation cohort: AUC 0.957 (95%CI 0.884–0.990), sensitivity 100%, specificity 91.1%, accuracy 93.4%, PPV 80%, NPV 100%;	-	AUCs 0.860–1, sensitivity 75–100%, specificity 87.5–100%, accuracy 88–100%, PPV 71.4–100%, NPV 88.9–100%
					Test cohort: AUC 0.923 (95% CI 0.838–0.971), sensitivity 82.1%, specificity 95.8%, accuracy 90.8%, PPV 92%, NPV 90.2%		
Laino (2023) [20]	Retrospective single centre	378	Hepatectomy for any indication	-	Combined preoperative + intraoperative + radiomics model AUC 0.802, sensitivity 77.5%, specificity 72.3%,	-	-
					accuracy 73.2%, PPV 39.2%, NPV 93.2%		
Zhong (2023) [21]	Prospective single centre	345	Treatment-naïve HCC patients	RF-based	Cross-validation: AUC 0.867 (95% CI 0.787–0.947);	First-order radiomics	-
					Test cohort: AUC 0.822 (95% CI 0.720–0.898), accuracy 0.750, sensitivity 0.704, specificity 0.773, PPV 0.612, NPV 0.836		
Xu (2023) [22]	Retrospective single centre	265	Hemihepatectomy irrespective of etiology	DenseNet-based CNN	AUC 0.7927, accuracy 84.15%, sensitivity 71.43%, specificity 89.66%	-	-
Lu (2022) [23]	Retrospective multi-institutional	871	HBV-related HCC patients	ANN	AUC 0.864 (95% CI 0.801–0.913), sensitivity 82.6%, specificity 74.6%, PPV 35.2%, NPV 96.3%	TBil, AST, ICGR-15	AUC 0.872 (95% CI 0.831–0.906), sensitivity 87.2%, specificity 77.7%, PPV 39%, NPV 97.4%
Wang (2022) [24]	Retrospective multi-institutional	875	Treatment-naïve HCC patients	LightGBM	Internal validation: AUC 0.870 (95% CI, 0.791–0.950), sensitivity 100%, specificity 64.4%, accuracy 70.5%, PPV 36.6%, NPV 100%;	PLT count, age, Creatinine	-
					Testing cohort: AUC 0.822 (95% CI 0.755–0.888), sensitivity 87.5%, specificity 64.4%, accuracy 69.7%, PPV 42.2%, NPV 94.6%		
Mai (2020) [25]	Retrospective single centre	353	Hemihepatectomy in treatment-naïve HCC patients	ANN	AUC 0.876 (95% CI: 0.801–0.950), sensitivity 95.2%, specificity 65.7%, PPV 45.51%, NPV 97.78%	sFLR, TBil, PLT count	-

## Data Availability

Data sharing is not applicable, since no new data was generated.

## Supplementary Materials

The following supporting information can be downloaded at https://www.mdpi.com/article/10.3390/medicina62020237/s1: Table S1: Complete search strategy.

## Abbreviations

The following abbreviations are used in this manuscript:

PHLF	Posthepatectomy liver failure
ML	Machine learning
AI	Artificial intelligence
ISGLS	International study group of liver surgery
MELD	Model for end-stage liver disease
ALBI	Albumin–bilirubin score
FIB-4	Fibrosis-4 index
AUC	Area under the receiver operating characteristic curve
SHAP	SHapley Additive exPlanations
MeSH	Medical Subject Headings
HCC	Hepatocellular carcinoma
ANN	Artificial neural network
sFLR	Standardized future liver remnant
TBil	Total bilirubin
PLT	Platelet count
PPV	Positive predictive value
NPV	Negative predictive value
BERT	Bidirectional encoder representations from transformers
POD1	Postoperative day 1
PT-INR	Prothrombin time/international normalized ratio
HBV	Hepatitis B virus
LightGBM	Light gradient boosting machine
APRI	AST to platelet ratio index
CONUT	Controlled nutritional status score
NSQIP	National Surgical Quality Improvement Program
Na	Sodium
XGBoost	Extreme gradient boosting
DCA	Decision curve analysis
CT	Computed tomography
MRI	Magnetic resonance imaging
AEM	Average ensemble model
RF	Random forest
CNN	Convolutional neural network
LR+	Positive likelihood ratio
LR−	Negative likelihood ratio
