# Supplementary material for: Machine Learning-Driven Risk Prediction Models for Posthepatectomy Liver Failure: A Narrative Review
Source: Medicina (Kaunas). 2026 Jan 23;62(2):237. doi: 10.3390/medicina62020237 (PMC12942370; doi:10.3390/medicina62020237)
Supplement: Supplementary file 1 [file medicina-62-00237-s001.zip › medicina-4083142-supplementary.pdf]

*Supplementary Table S1. Complete search strategy.*

**PubMed/Medline**

(''posthepatectomy liver failure'' OR PHLF OR "Hepatectomy/adverse effects"[Mesh]) AND ("prediction model" OR algorithm OR "artificial intelligence" OR AI OR "machine learning" OR ML OR "deep learning" OR "Machine Learning Algorithms"[Mesh] OR "random forest" OR "gradient boosting" OR "neural network"). Filters: 2010-2025, English --> 188 hits

**Scopus**

TITLE-ABS-KEY ((“machine learning" OR "artificial intelligence" OR "deep learning" OR "random forest" OR "gradient boosting" OR "neural network”) AND (“posthepatectomy liver failure" OR PHLF OR (“liver surgery" AND (outcome* OR complication*)) OR (hepatectomy AND (outcome* OR complication*)))) AND PUBYEAR > 2009 AND PUBYEAR < 2027 AND (LIMIT-TO (LANGUAGE, "English”)) --> 288 hits

**Web of Science Core Collection**

("machine learning" OR " artificial intelligence" OR "deep learning" OR "random forest" OR "gradient boosting" OR "neural network*") AND ("posthepatectomy liver failure" OR PHLF OR "liver surgery" OR hepatectom*) AND (prediction OR risk OR algorithm OR model) (Topic) and 2010-2025 (Publication Years) and English (Languages) --> 312 hits
